# Sequential events of apoptosis involving docetaxel, a microtubule-interfering agent: A cytometric study

**DOI:** 10.1186/1471-2121-7-6

**Published:** 2006-01-26

**Authors:** Francesco Fabbri, Silvia Carloni, Giovanni Brigliadori, Wainer Zoli, Rosa Lapalombella, Marina Marini

**Affiliations:** 1 Department of Medical Oncology, Morgagni-Pierantoni Hospital, Via Forlanini 34, 47100 Forlì, Italy; 2 Department of Histology, Embryology and Applied Biology, University of Bologna, Via Belmeloro 8, 40126 Bologna, Italy

## Abstract

**Background:**

Despite the great advances in the understanding of programmed cell death, little attention has been paid to the sequence of the events that characterise it. In particular, the course of apoptotic events induced by microtubule-interfering agents such as taxanes is poorly understood. In order to increase such knowledge, we studied a number of independent biochemical and cytological modifications using cytometric methods in a bladder cancer cell line treated with the second generation taxane, docetaxel.

**Results:**

Within a few hours, drug treatment had induced mitochondrial membrane transition, cell shrinkage and a decrease in granularity. Cell cycle was almost completely blocked in G_2_/M phase within 24 hours. The hypodiploid peak started to become prominent 48 hours after the treatment. At the same time, the appearance of a DNA ladder demonstrated caspase-dependent chromatin fragmentation. Concurrently, specific cell surface modifications took place, involving at first glycoprotein syalilation and later phospholipid asymmetry. DNA fragmentation was subsequently detected by TUNEL assay. Over time, cell membranes became permeable to propidium iodide. A very similar time-course of apoptotic events was found after treatment of a myelomonocytic cell line with the same drug.

**Conclusion:**

After discussing some characteristics of the methods employed and their limitations, a succession of apoptotic events over time is suggested, in which the collapse of mitochondrial transmembrane potential (Δψm) is the earliest sign of apoptosis.

## Background

Apoptosis is the most widespread form of cell death, characterised by distinguished biochemical and morphological events triggered by signalling cascades and finely regulated by the products of oncogenes, tumour suppressor genes, and apoptosis-specific genes. It has proven useful to distinguish two main phases in apoptotic cell death: *induction *and *execution*. Induction is controlled by a wide range of mechanisms that involve either surface (extrinsic) signalling and subsequent signal transduction mechanisms, or internal (intrinsic) pathways leading to the release of mitochondrial components. Both pathways converge in common events during the execution phase. These events specifically characterise apoptotic cell death and lead to the successful phagocytosis of cell corpses, still surrounded by leak-proof membranes [1,2].

Both basic and clinically oriented studies require reliable evaluation and quantification of apoptosis. In particular, most chemotherapeutic drugs trigger apoptosis in actively dividing cells. A good deal of information about their efficacy and their therapeutic index may be obtained by the identification of cell death mode (apoptosis *vs*. necrosis), quantification of dead cells, and by understanding the effect of the drug on the cell cycle.

The unique changes that characterise the apoptotic process provide several features that permit the recognition and quantitation of apoptotic cell death by cytometric methods [3]. These methods have two important advantages in that they are quantitative and allow the possibility of evaluating multiple parameters, including cell cycle status. Some of them exploit specific features that are a hallmark of apoptosis, such as DNA degradation – as revealed by reduced binding of the intercalating fluorescent dye, propidium iodide [4], by the labelling of DNA strand breaks [5], or by the redistribution of membrane phospholipids, which can be detected by tagged fluorochromes [6]. A common parameter for distinguishing between apoptosis and necrosis is the integrity of the cell membrane, which blocks the entry of propidium iodide in non-permeabilised cells [7]. In the course of signal transduction that leads to apoptosis, mitochondria may release pro-apoptotic molecules, a process accompanied by loss of the transmembrane potential (Δψm) that can be detected by specific fluorescent markers [8]. The centralrole played by mitochondria in the control of both intrinsic and extrinsic apoptotic pathways is well known [9].

The aim of the present study was to determine the temporal succession of the main events that characterise taxane-induced apoptosis in a cancer cell line. Taxanes induce apoptosis by interfering with microtubule dynamics, in particular by preventing tubulin depolymerisation. Actively dividing cells are thus induced to undergo apoptosis, triggered by bcl-2 phosphorylation and involving the activation of the c-Raf-1/Ras or the p53/p21WAF1/CIP1 signalling pathway [10]. In epithelial cells, taxol induces a cyclosporin A (CsA)-sensitive decrease in ψm and a Ca^2+ ^efflux from the mitochondria [11]. It has also been suggested that changes in the microtubule structure induced by taxanes may be responsible for opening the mitochondrial permeability transition pores [12].

In this and in most drug-induced apoptotic processes, the release of mitochondrial components is a requisite for the activation of caspases, which are apoptosis-specific proteases [13]. DNA fragmentation and most of the biochemical and morphological changes that characterise apoptosis then take place under the control of effector caspases. However, the order of events has seldom been investigated. We therefore evaluated a number of supposedly independent apoptotic events and their temporal succession in an attempt to determine which are actually independent from the other.

A secondary aim of the study was to compare multiple cytometric methods to study the apoptotic process and acquire information that can be used as a guideline for the choice of the most suitable parameter(s) for different experimental systems. In particular, since some methods actually assess the same event, we will discuss possible discrepancies in results. These parameters can be employed to assess the execution phase of apoptosis, which is believed to take place in a stereotyped way, independently of the apoptotic trigger [14].

## Results

### Antiproliferative and cytocidal effects

**Figure 1:**
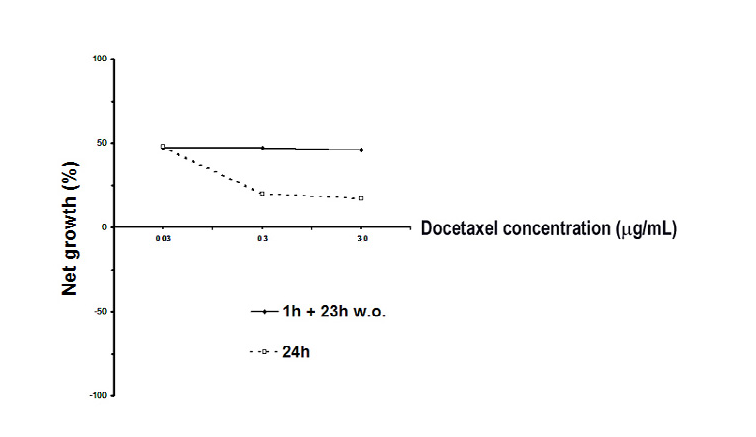
Cytotoxic and antiproliferative activity of docetaxel in the HT1376 bladder cancer cell line, evaluated by Sulphorhodamine B assay. The percentage of cell growth is defined in the *Methods *section. Continuous line: 1-hour treatment with docetaxel followed by a 23-hour wash-out. Dotted line: 24-hour continuous treatment. Experiments were run in octuplet, and each experiment was repeated three times. Standard errors were below 5%

Docetaxel exerted an antiproliferative effect on HT 1376 tumour cell line at a concentration as low as 0.03 μg/mL (Fig. 1). In fact, after either 24 hours' continuous exposure to the drug or a 1-hour treatment followed by a 23-hour wash-out, cell growth was greatly reduced compared to that of unexposed cells. No dose-response relationship was observed in the wash-out experiments, whereas continuous exposure to docetaxel (DOC) concentrations higher than 0.03 μg/mL resulted in a more pronounced cytostatic effect, as shown by a further decrease in cell growth as drug concentrations increased. One possible interpretation is that DOC-induced growth arrest was maximal even at the lowest concentration tested, but affected, during the 1-hour exposure, only a fraction of the cell population. Notably, SRB test was carried out for 24 hours only. It is likely that, if carried out for a longer time, we would have observed a cytocidal effect, as suggested by results from the other experimental procedures presented in our study.

As the amount of DOC-induced necrosis was negligible (see below), further experiments were carried out at the highest DOC concentration (3 μg/mL, corresponding to 3.7 μM), in order to achieve more clear-cut results. Moreover, this concentration corresponds to the standard plasmatic concentration achieved in *in vivo *treatments of patients [15].

### Cell cycle kinetics, apoptosis and scatter variation analysis

**Figure 2:**
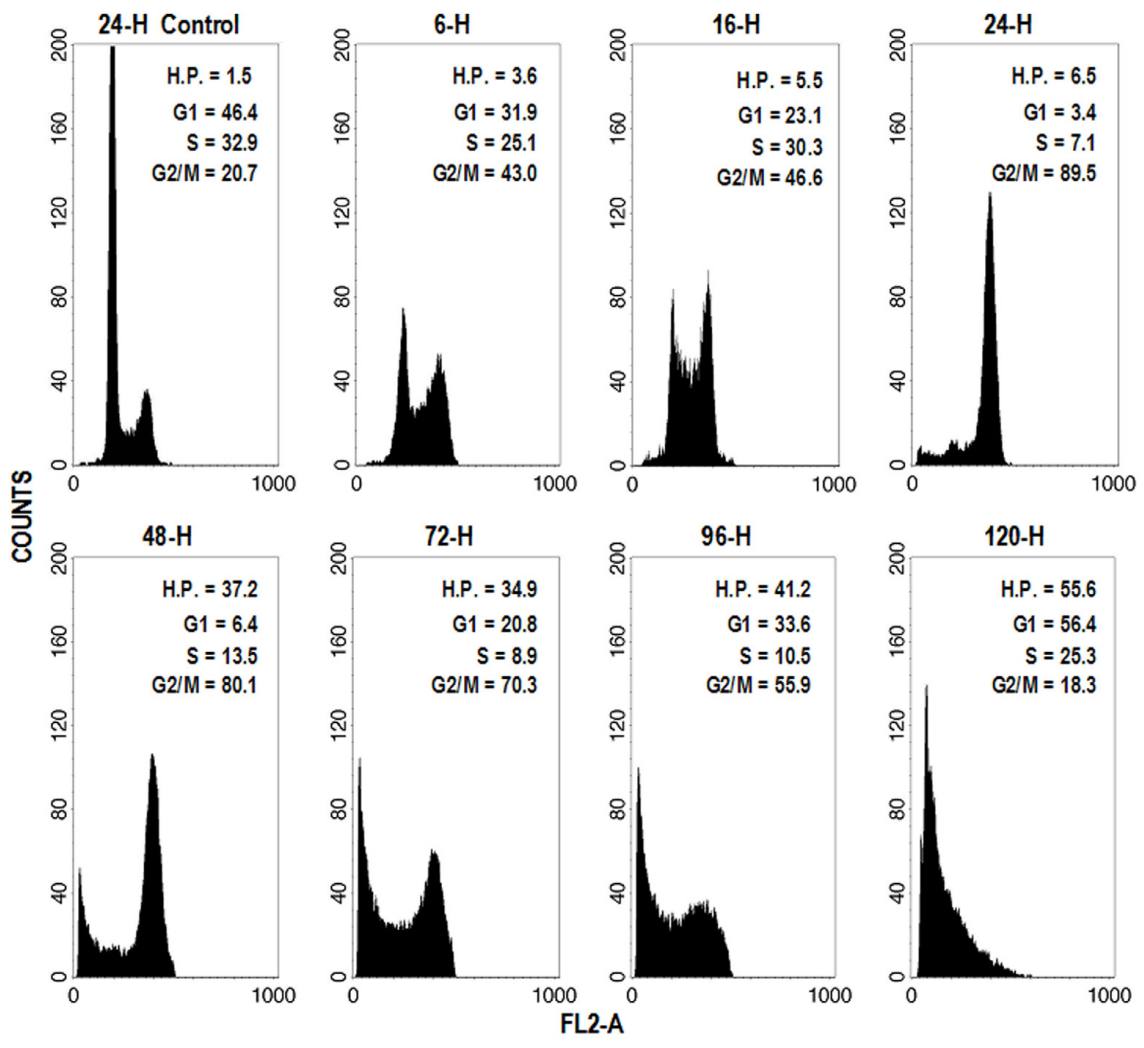
Cell cycle distribution of HT1376 bladder cancer cell line after a 1-hour treatment with 3 μg/mL docetaxel followed by different wash-out times. Cells were permeabilised and stained with propidium iodide, as described in the *Methods *section. Histograms show a representative experiment.

As shown in Fig. 2, DOC (3 μg/mL) induced a dramatic accumulation of HT 1376 tumour cells in the G_2_/M compartment. Six hours after the beginning of treatment, the G_0/1 _peak was already markedly reduced and by 24 hours it had almost disappeared. The G_2_/M phase arrest was not released for the 95 hours of recovery that followed DOC treatment, whereas a hypodiploid sub-G_0/1 _peak was visible after 48 hours and gradually became more prominent, thus indicating a massive apoptotic process. The apparent increase in the G_0/1 _compartment observed subsequently was due to the progressive loss of propidium iodide (PI) stainability in cells undergoing apoptosis in the G_2_/M phase. This demonstrates that DNA fragmentation, affecting PI binding, occurs in G_2_-arrested cells.

**Figure 3:**
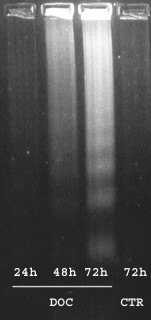
DNA gel electrophoresis of docetaxel-treated (lanes 1–3) and control (lane 4) HT1376 cells. DNA from HT1376 cells treated for 1 hour with 3 μg/mL docetaxel and cultured for a further 16, 24 and 48 hours was extracted as detailed in the *Methods *section, then separated in 1.5% agarose gel electrophoresis. DNA from a 72-hour control (untreated) culture was prepared in the same way.

Agarose gel DNA electrophoresis (Fig. 3) shows that chromatin underwent internucleosomal cleavage within 48 hours of treatment with DOC, as demonstrated by the formation of the characteristic ladder pattern of DNA migration.

**Figure 4:**
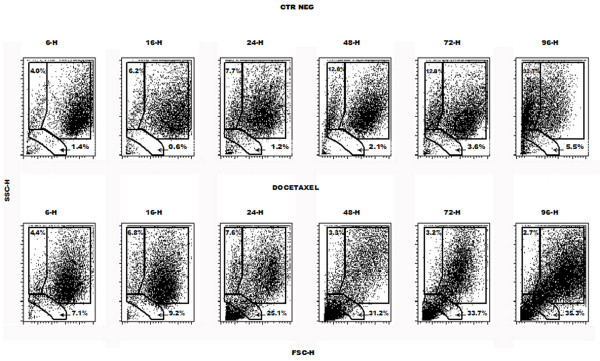
Variation in forward and side scatter of HT1376 bladder cancer cell line after a 1-hour treatment with 3 μg/mL docetaxel followed by different wash-out times. Diagrams show a representative experiment. Owing to a decrease in size and granularity, apoptotic cells progressively accumulate in the lower left area of the diagram, whereas the lower left corner corresponds to cell debris.

Forward and side scatter variation analysis (Fig. 4) shows a reduction in cell volume and granularity in DOC-treated cells as early as 16 hours after the beginning of treatment. Shrinkage also occurred in controls, starting after about 48 hours of culture. This may be due to the fact that, by that time, a reduction in growth rate was evident, as a consequence of crowding (table 1). Nevertheless, after control cultures were carried out for 96 hours without changing the medium or splitting the flasks, apoptosis amounted to about 10% and necrosis to a further 8% of cells (table 1). However, only DOC-treated cells showed a reduction in both forward and side scatter, a characteristic feature of apoptotic cells. The percentage of cells with these features gradually increased, reaching a plateau at 48 hours.

**Table 1:** Percentage of apoptotic cells: comparison of results obtained with different methods in HT 1376 cells

***UNTREATED CULTURES***																												
METHOD	CULTURE TIME																											

	**6 hrs**				**16 hrs**				**24 hrs**				**48 hrs**				**72 hrs**				**96 hrs**				**120 hrs**			

Cell Cycle	HP	G_0/1_	S	G_2_/M	HP	G_0/1_	S	G_2_/M	HP	G_0/1_	S	G_2/_M	HP	G_0/1_	S	G_2_/M	HP	G_0/1_	S	G_2_/M	HP	G_0/1_	S	G_2_/M	HP	G_0/1_	S	G_2_/M
(PI staining)	1.0	42.6	37.4	20.0	1.0	44.0	35.4	20.6	2.0	47.9	30.5	21.6	2.6	60.5	31.0	8.5	2.1	64.5	25.7	9.8	3.3	70.1	19.3	10.6	5.6	78.1	12.3	9.6
																												

	**6 hrs**				**16 hrs**				**24 hrs**				**48 hrs**				**72 hrs**				**96 hrs**							

JC-1	1.5				2.0				3.5				2.5				4.6				5.1							
Low FSC area	4.4				5.6				8.9				11.8				10.8				30.7							
Low FSC/SSC area	1.7				0.4				0.8				2.3				3.0				4.5							
MAA+PI staining	**E**	**L**	**N**		**E**	**L**	**N**		**E**	**L**	**N**		**E**	**L**	**N**		**E**	**L**	**N**		**E**	**L**	**N**					
	N.E.	N.E.	N.E.		N.E.	N.E.	N.E.		0.5	0.6	5.4		1.5	1.3	7.2		3.1	3.4	10.6		5.0	5.8	10.8					
AnnV+PI staining	**E**	**L**	**N**		**E**	**L**	**N**		**E**	**L**	**N**		**E**	**L**	**N**		**E**	**L**	**N**		**E**	**L**	**N**					
	N.E.	N.E.	N.E.		N.E.	N.E.	N.E.		2.9	0.7	4.5		2.0	3.2	5.1		1.2	4.7	7.3		3.1	5.9	8.2					
TUNEL	N.E.				N.E.				0.5				0.8				1.6				1.5							

***DOCETAXEL-TREATED CULTURES***																												

METHOD	CULTURE TIME																											

	**6 hrs**				**16 hrs**				**24 hrs**				**48 hrs**				**72 hrs**				**96 hrs**				**120 hrs**			

Cell Cycle	HP	G_0/1_	S	G_2_/M	HP	G_0/1_	S	G_2_/M	HP	G_0/1_	S	G_2/_M	HP	G_0/1_	S	G_2_/M	HP	G_0/1_	S	G_2_/M	HP	G_0/1_	S	G_2_/M	HP	G_0/1_	S	G_2_/M
(PI staining)	3.1	30.9	27.5	41.6	4.9	25.7	29.0	45.3	5.5	3.1	9.1	87.8	35.5	5.9	12.3	81.8	33.3	19.8	7.9	72.3	39.0	31.4	10.9	57.7	53.5	57.1	23.4	19.5
																												

	**6 hrs**				**16 hrs**				**24 hrs**				**48 hrs**				**72 hrs**				**96 hrs**							

JC-1	3.0				10.4				24.5				53.6				60.0				91.3							
Low FSC area	5.4				7.4				6.6				2.2				2.3				3.3							
Low FSC/SSC area	6.5				8.5				24.2				29.6				32.5				33.6							
MAA+PI staining	**E**	**L**	**N**		**E**	**L**	**N**		**E**	**L**	**N**		**E**	**L**	**N**		**E**	**L**	**N**		**E**	**L**	**N**					
	N.E.	N.E.	N.E.		N.E.	N.E.	N.E.		0.5	0.7	9.9		28.1	25.1	12.5		19.0	39.6	12.9		21.2	42.7	15.6					
AnnV+PI staining	**E**	**L**	**N**		**E**	**L**	**N**		**E**	**L**	**N**		**E**	**L**	**N**		**E**	**L**	**N**		**E**	**L**	**N**					
	N.E.	N.E.	N.E.		N.E.	N.E.	N.E.		6.5	0.9	5.1		14.7	15.9	7.5		27.7	43.4	8.3		21.7	56.5	9.9					
TUNEL	N.E.				N.E.				4.7				9.3				28.6				70.4							

Top table: untreated (control) HT1376 bladder carcinoma cultures. Bottom table: HT1376 bladder carcinoma cultures were treated for 1 hour with 3 μg/mL docetaxel, after which the drug was washed out and the cells were further cultured. Figures are percentages of cells with the indicated characteristics. Data are the average of 3 independent experiments, with errors below 10%. For details, see the Methods section. N.E. = Not Examined; HP = Hypodiploid Peak; JC-1 = cells undergoing Δψ_m _transition; E = early apoptosis, i.e. MAA+/PI- or AnnV+/PI-; L = late apoptosis, i.e. MAA+/PI+ or AnnV+/PI+; N = necrosis, i.e. MAA-/PI+ or AnnV-/PI+

### Mitochondrial pore transition

**Figure 5:**
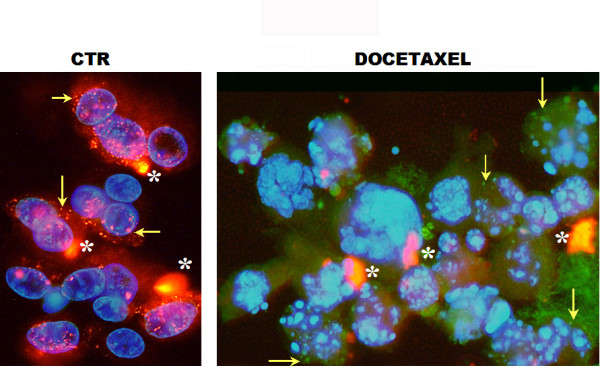
Double staining of nuclei (DAPI blue) and mitochondria (JC-1). Left panel: control cells with intact nuclei, where JC-1 aggregates inside healthy mitochondria and fluoresces red (arrows). Right panel: apoptotic HT1376 bladder cancer cells with fragmented nuclei, where the monomeric form of JC-1 diffuses into the cytoplasm and fluoresces green (arrows). Cells in the right panel were treated with docetaxel (1 hour followed by a 96-hour wash-out). The aggregation of JC-1 in the mitochondria is driven by the transmembrane potential. Aggregated extracellular dye deposits (stars) are artifactual.

In cells with high mitochondrial membrane potential, the fluorochrome JC-1 formed dye aggregates, which fluoresced red. Cells with low potential contained monomeric JC-1 and fluoresced green. Thus, these reversible changes in aggregation helped to identify cell status, as seen in Fig. 5, where DAPI staining reveals the nuclear fragmentation in apoptotic cells, which showed a loss of mitochondrial membrane potential.

Cytometric analysis shows that treatment of HT1376 cells with DOC caused an early transition of JC-1 fluorescence, with a shift toward green fluorescence that was detected as early as 16 hours after treatment. The percentage of cells with impaired mitochondrial membrane potential steadily increased, reaching 90% of cells 96 hours after treatment (table 1).

For comparison purposes, the U937 myelomonocytic cell line was treated for 1 hour with the same concentration of DOC (3 μg/mL). The drug was then washed out and the cells were cultured for a further 15, 23, or 47 hours, stained with JC-1 and examined by cytometry. The mitochondrial pore transition was found to occur in U937 at the same time as in HT 1376 cell line, i.e. as early as 15 hours after treatment (table 2).

**Table 2:** Percentage of apoptotic cells: comparison of results obtained with different methods in the U937 myelomonocytic cell line

Method	Culture time											
	**16 hrs**			**24 hrs**			**48 hrs**			**Untreated controls, 48 hrs**		

JC-1	15.6			27.3			56.4			4.0		
MAA + PI staining	E	L	N	E	L	N	E	L	N	E	L	N
	18.4	2.0	4.6	23.8	4.4	8.4	38.0	29.0	6.5	1.0	1.5	5.6
Ann V + PI staining	E	L	N	E	L	N	E	L	N	E	L	N
	19.5	6.5	1.2	28.7	10.1	3.5	40.9	22.0	2.8	2.9	1.5	1.8

U937 myelomonocytic cells were treated for 1 hour with 3 μg/mL docetaxel, after which the drug was washed out and cells were further cultured. Data are the average of 2 experiments, with nearly identical results. For details, see the Methods section.

JC-1 = cells undergoing Δψ_m _transition; E = early apoptosis, i.e. MAA+/PI- or AnnV+/PI-; L = late apoptosis, i.e. MAA+/PI+ or AnnV+/PI+; N = necrosis, i.e. MAA-/PI+ or AnnV-/PI+

To evaluate the effect of CsA-induced blockage of Ca^2+ ^efflux from mitochondria, HT 1376 cells were pre-incubated with 1 μM cyclosporin A (CsA) for 30 min before treatment with DOC. CsA was washed out at the same time as DOC, i.e. after a 1-hour exposure. Cells were stained with JC-1 or AnnV + PI, as detailed in the *Methods *section, immediately after the drug wash-out (1 hr) and after a 16- and 24-hour incubation. Results showed that CsA treatment did not affect the mitochondrial Δψ variation (table 3).

**Table 3:** Effect of pre-incubation with CsA on DOC-treated HT 1376 cells.

	**1 h**			**16 hrs**			**24 hrs**		
**Control**									

JC-1	1.0			2.1			4.7		
AnnV + PI staining	E	L	N	E	L	N	E	L	N
	1.9	0.5	1.5	2.1	0.9	3.1	2.8	1.1	5.0

**DOC-treated**									

JC-1	1.0			9.5			24.0		
AnnV + PI staining	E	L	N	E	L	N	E	L	N
	2.1	0.9	2.0	5.0	0.5	3.5	6.0	2.5	5.5

**CsA + DOC treated**									

JC-1	1.0			5.2			23.1		
AnnV + PI staining	E	L	N	E	L	N	E	L	N
	2.0	0.7	2.5	5.0	1.1	4.2	5.0	4.3	8.4

HT 1376 bladder carcinoma cells were pre-incubated for 30 min with 1 μM CsA, after which 3 μg/mL docetaxel was added. After a 1-h treatment, both drugs were washed out and cells were further cultured. Data are percentages of cells with the indicated staining characteristics. For details, see the Methods section.

JC-1 = cells undergoing Δψ_m _transition; E = early apoptosis, i.e. AnnV+/PI-; L = late apoptosis, i.e. AnnV+/PI+; N = necrosis, i.e. AnnV-/PI+

### Necrosis

Double staining with MAA lectin or Annexin V (AnnV) and PI allowed for the evaluation of necrosis (tables 1 and 2). After 96 hours of culture, DOC-treated HT 1376 cells stained with PI alone (primary necrosis) accounted for only 16% of the total cell population, a value just slightly higher than that of control (untreated) cells. The percentage of DOC-treated PI+ U937 cells was also negligible. This demonstrates that DOC induces cell death largely by apoptosis.

Our results clearly show that cells labelled by an apoptosis marker, such as AnnV, also tend to become permeable to PI due to the loss of membrane integrity (tables 2 and 3).

### MAA lectin

DOC-treated HT1376 cells developed affinity for MAA lectin 24 to 48 hours after exposure to the drug (table 1). When evaluated 48 hours after DOC treatment, more than 50% of cells were found to be MAA+, but about half of them had already lost their membrane integrity, undergoing secondary necrosis and becoming permeable to propidium iodide. Over time, whilst more and more cells underwent apoptosis and formerly apoptotic cells acquired PI permeability, the percentage of MAA+/PI- cells remained stable, whereas that of MAA+/PI+ increased, thus showing that MAA binding initially occurred in apoptotic rather than necrotic cells.

DOC-treated U937 cells were also recognized by MAA lectin, but at an earlier culture times (16 hours) than HT1376. The percentage of MAA+ U937 cells steadily rose as culture time increased, and a subset of MAA+ cells became PI+.

### Phosphatidylserine exposure

Forty-eight hours after DOC treatment, HT1376 cells started displaying AnnV-staining above control levels (table 1), but the percentage of AnnV+ cells was lower than that of MAA+ cells, thus suggesting that phosphatidylserine (PS) exposure occurs later than carbohydrate changes affecting MAA binding. Over time, as already seen for MAA+ cells, the percentage of AnnV+/PI- cells remained stable, whereas that of AnnV+/PI+ increased. AnnV+ cells, whether PI- or PI+, outnumbered MAA+ cells at 72 and 96 hours, thus indicating that a subset of AnnV+ cells did not bind MAA lectin.

DOC-treated U937 cells also displayed AnnV staining, with a time course and cell percentages similar to those of MAA staining (table 2). As culture time increased, a subset of AnnV+ U937 cells became PI+. The fact that the time course of MAA staining and the percentage of MAA+ cells paralleled that of AnnV in both cell types reinforces the hypothesis that MAA is a marker of apoptosis, detecting the same cell subset as AnnV.

As the AnnV staining method requires incubation at room temperature, at variance with the staining method for JC-1, we performed an experiment to evaluate whether a mock incubation (no additions) mimicking JC-1 staining at 37°C before staining with AnnV at room temperature would alter the amount of apoptosis recognized by AnnV. No variation in the percentage of AnnV+ apoptotic cells was observed after a ten-minute incubation at 37°C. Results are not reported for the sake of brevity.

### Cell count and viability

To confirm data on cell cycle kinetics and to evaluate the percentage of viable and non viable cells using an independent method, an aliquot of control and DOC-treated HT 1376 cells was stained with trypan blue and counted at different culture times. Results are shown in table 4. Newly-seeded control cells displayed a 1.85-fold increase in number within the first 24 hours of culture. The doubling time subsequently increased as a result of crowding, but total cell count was about 5-fold higher than the initial one after 4 days of culture. Conversely, cell growth was found to be impaired in DOC-treated cells with respect to the first determination (1.42-fold increase within the first 24 hours). DOC-treated HT 1376 cells completed about one round of duplication within the first 48 hours, but did not show further growth. When evaluated at later times, a slight reduction in the total cell count was seen (about 23% after 96 hours of culture), probably due to the fragmentation of apoptotic cells. These data confirm that treatment with DOC results in cell cycle inhibition and in a subsequent induction of cell death, as already shown by the SRB test and by cell cycle kinetics (Fig. 1, 2).

**Table 4:** Cell count and viability

**Untreated HT1376 cells**	**6 hrs**	**16 hrs**	**24 hrs**	**48 hrs**	**72 hrs**	**96 hrs**
Total cell count (10^6^/mL)	5.625	9.375	10.42	17.3	23.625	27.25
% Viable cells	97.0	98.1	95.5	93.0	94.5	91.3
% Dead cells	3.0	1.9	4.5	7.0	5.5	8.7

**DOC – treated HT1376 cells**	**6 hrs**	**16 hrs**	**24 hrs**	**48 hrs**	**72 hrs**	**96 hrs**

Total cell count (10^6^/mL)	4.281	6.887	7.706	10.545	9.647	8.557
% Viable cells	96.5	94.8	95.0	70.3	46.7	32.6
% Dead cells	3.5	5.2	5.0	29.7	53.3	67.4

HT 1376 bladder carcinoma cells were stained with Trypan Blue and evaluated with a hemocytometer, as described in Methods. Data reported in the table are from one representative experiment. Counting error was ± 0.15 × 10^6 ^cells. The count was repeated once, with virtually identical results.

Trypan blue stained only non viable cells, i.e. cells that have lost their membrane integrity. On a time-by-time basis, the percentage of non viable cells reported in table 3 corresponds to that obtained with PI staining reported in table 1, i.e. the sum of AnnV+/PI+ and AnnV-/PI+ cells.

These data suggest that, using cytofluorimetric methods alone, a comprehensive and reliable picture of both cell cycle kinetics and cell viability can be obtained.

### DNA strand breaks

TUNEL staining of HT1376 apoptotic cells, indicative of massive DNA strand breaks, was not observed until 72 hours after DOC treatment. The percentage of TUNEL+ cells had markedly increased after 96 hours of culture, approaching that of AnnV+ cells (table 1). It is noteworthy that DNA strand breaks were detected at earlier times with other methods, such as PI staining (Fig. 2) and DNA ladder formation assay (Fig. 3).

## Discussion

Despite the considerable amount of information on apoptosis in tumour cell lines acquired from cytometry-based studies, little attention seems to have been paid to the time course of the main events that characterise the apoptotic process, and in particular those induced by taxanes.

Earlier studies established that phosphatidylserine exposure to the outer membrane leaflet occurs simultaneously with chromatin condensation [16] and precedes DNA fragmentation [17]. Other membrane changes, as shown by an increase in Merocyanin 540 binding of apoptotic cells [18-20], may not be independent of the phosphatidylserine contribution to the membrane lipid composition [21]. On the other hand, AnnV staining itself may be lacking in certain apoptotic cell populations [20]. These findings underline the need to evaluate multiple parameters when assessing cell death. A number of studies have addressed the issue of mitochondrial involvement in the apoptotic process by evaluating Δψm decrease with different dyes [22-24] inan attempt to establish a temporal – and possibly – causal relationship within the events that characterise apoptosis. It appears that the bcl-2 content of the cells may be related to the timing of mitochondrial involvement [25]. This consideration is particularly important in taxane-induced apoptosis, where bcl-2 is involved in the cell death induction mechanisms as well as in drug resistance [26-28].

Taxanes and their derivatives inhibit microtubule depolymerisation, thus preventing cell cycle completion [29] and thus causing apoptotic cell death. A similar effect is also caused by drugs that inhibit microtubule polymerisation, such as vinblastine and vincristine. Apoptosis triggered by agents that disrupt microtubule organisation is associated with bcl-2 phosphorylation and is independent of p53 involvement, at variance with apoptosis due to DNA-damaging agents [30]. Despite these differences, both microtubule disruption and DNA damage activate the intrinsic pathway of cell death, causing the release of mitochondrial components and the formation of apoptosomes. In both cases, the triggering mechanism is believed to involve changes in the amount and/or activity of anti- and pro-apoptotic members of the bcl-2 family.

Conversely, it has been shown that administration of paclitaxel – the prototype for taxane compounds – causes an almost immediate increase in cytosolic Ca^2+^, associated with a rapid decline in mitochondrial membrane potential, presumably due to the opening of the permeability transition pores (mPTPs) [11,31]. The rapidity of this event apparently rules out the participation of mechanisms controlling microtubule assembly status, thus suggesting a pathway of death induction different from that previously reported [30]. However, it is recognised that loss of Δψm and variations in [Ca^2+^]i may be transient and do not necessarily lead to apoptosis. In particular, mPTPs have two conformations, one characterised by low- and the other by high-conductance state [32]. The opening of the former is reversible, allows the release of small molecules only – such as calcium ions – and may be involved in the rapid [Ca^2+^]i changes described by other authors [11]. In contrast, late changes in Δψm, such as those observed by us 16 hours after drug treatment, are likely to involve the high-conductance mPTP, which is responsible for the release of apoptosis-inducing compounds. The delayed change in Δψm is not apparently affected by prior exposure to CsA (table 3). However, it is obvious that cell cycle is already deeply affected as early as 6 hours after the end of drug treatment (Fig. 2). Thus, a more realistic view puts the mitochondrial involvement in apoptosis downstream of paclitaxel-induced G_2_/M arrest. Within this cell-cycle phase, microtubule impairment may activate mTOR kinase, thus affecting bcl-2 phosphorylation and its subsequent proteasome-dependent degradation [33]. The data on the action/activity of CsA presented in the present study are only preliminary and the role played by Ca^2+ ^variation in this process will be the object of future investigation.

Whilst the time course of early events has yet to be defined, its eventual clarification will provide new insight into the mechanism whereby taxanes *induce *cell death. Moreover, other well-studied events that belong to the *execution *phase of apoptosis have not yet been placed in a precise time setting. These include phosphatidylserine exposure to the outer cell surface, DNA cleavage, and loss of membrane integrity. Changes in surface glycosylation are less well understood and have been included here because they currently being studied by the authors (Marini et al., manuscript in preparation). Our results suggest that these changes are not peculiar to a given cell type and potentially represent a new marker of apoptosis. They would seem to occur about the same time as PS exposure to the outer surface and may be related to it.

As described in the present work, apoptotic events in the HT1376 bladder cancer cell line treated with DOC would appear to occur in the following order: 1) cell cycle arrest; 2) loss of mitochondrial membrane potential; 3) cell shrinkage and decrease in granularity; 4), DNA fragmentation (hypodyploid peak and electrophoretic laddering); 5) and 6) change in syalilation of surface proteins and loss of surface phospholipid asymmetry; 7) loss of membrane integrity (cells become PI+); 8) further DNA fragmentation (TUNEL). The above outlined sequence of events takes into account intrinsic limitations in cytometric methods, which include a lesser degree of sensitivity in comparison with biochemical determinations and the variability in drug response within an asynchronous cell population. In fact, it must be stressed that all the cells do not die simultaneously after drug treatment because death is dependent on factors such as the cell cycle status and the individual energy level. Moreover, in most artificial systems, e.g. culture flasks, cells dying by apoptosis are not phagocyted by competent cells and eventually lose their membrane potential and undergo secondary necrosis. Thus, within an asynchronously growing cell population, cycling cells, cycle-arrested cells, cells undergoing apoptosis and cells already undergoing secondary necrosis can all be found and will be sending out conflicting signals that require interpretation.

Data on DOC-treated U937 myelomonocytic cell lines were reported in the present work for comparison purposes and were not as complete as those obtained on HT 1376 bladder cancer cell line. Nevertheless, they suggest that Δψm is an early event in both cell types, that bear though different they may be, and that MAA marks the same cell subset as AnnV.

In permeabilised cells, DNA fragmentation results in decreased PI staining (i.e. cells appear to have a hypodiploid DNA content) [7]. Accordingly, this event appears to occur between 24 and 48 hours after drug administration. However, the onset of DNA fragmentation may occur earlier, as cells with duplicated DNA join the sub-G_0/1 _peak after passing through the S and G_0/1 _peaks. On the other hand, it is possible that, over time, the progressive increase in DNA fragmentation causes a marked loss in PI stainability, causing the hypodiploid peak to be underestimated, as suggested by comparing data with results obtained from unpermeabilised cells and from TUNEL staining, another index of DNA fragmentation. This technique, where 3' OH termini – resulting from DNA cleavage – are labeled with FITC-conjugated nucleotides in a reaction utilising exogenous terminal deoxynucleotide transferase (TdT), is apparently less sensitive than a decrease in PI stainability, possibly because it requires more extensive DNA cleavage. However, for longer culture times, TUNEL staining appears to give a better estimate of the percentage of apoptotic cells with respect to the evaluation of the hypodiploid peak. In fact, 96 hours after DOC treatment, the percentage of TUNEL+ cells was much higher than that of cells in the sub-G_0/1 _peak, and approximated that of AnnV+ cells.

The characteristic ladder revealed by gel electrophoresis of DNA from DOC-treated cells demonstrates that chromatin fragmentation did not occur randomly, but rather at internucleosomal sites. Neither high molecular weight DNA cleavage (as occurs during the initial stages of apoptosis), nor aspecific DNA fragmentation (following necrotic cell death), produce such a specific pattern of electrophoretic migration. The same applies to the formation of the hypodiploid peak [34].

Cell surface modifications were observed 24 hours after DOC treatment and over time extended to other cells (table 1). Due to its strong affinity to negatively charged phospholipids, AnnV binds phosphatidylserine as it becomes exposed to the outer cell surface, a common feature of cells undergoing apoptosis. Variations in cell surface carbohydrate composition can be identified by changes in lectin-mediated recognition. Apoptotic cells may change the oligosaccharide composition of their glycocalix, thus displaying a differential binding to some lectins. In particular, Maakia Amurensis lectin (MAA), which preferentially recognises galactose residues bound to α 2,3 sialylic acid [35], was found to bind to apoptotic cells rather than to their "healthy" counterpart (Marini et al, manuscript in preparation). The percentage of cells showing variations in the glycosylation of surface molecules initially increased more sharply than that of cells displaying phosphatidylserine on the outer membrane leaflet. However, over time the percentage of MAA+ cells did not reach that of AnnV+ cells. Studies are ongoing to further the understanding of these variations in surface syalilation and of their relevance in apoptosis. As already pointed out, the cell population appeared to acquire both MAA and AnnV markers before losing its membrane integrity (i.e. before becoming permeable to PI). In addition to such information, the percentages of apoptotic PI- and PI+ cells observed shows that the increase in TUNEL positivity (i.e. progression in DNA degradation) also occurred in cells that had lost membrane integrity.

Ninety-six hours after drug treatment, the near totality of cells displayed a decrease in transmembrane mitochondrial potential Δψ_m _that accompanied the efflux of pro-apoptotic molecules from the mitochondrion, as evaluated by the cationic fluorochrome JC-1 (table 1). This suggests that almost all of the DOC-treated cells eventually undergo apoptosis. Moreover, it supports the view hypothesis that mitochondrial events occur very early during cell culture and pave the way for both DNA cleavage and surface modifications. Furthermore, cytoskeleton modifications that cause cell shrinkage and loss of granularity (Fig. 4) may be subsequent to a loss of Δψ_m_, as suggested elsewhere [36].

In the present paper we compared cytofluorimetric with non cytofluorimetric methods of evaluation of cell death. The same conclusions about the cytotoxic and cytostatic effects of the drug were reached using the SRB assay, the dye-exclusion test and the cytofluorimetric evaluation of cell cycle. Apoptosis-induced cell surface and chromatin modifications can be assessed by both morphological evaluation and cytometry. However, cytometry appears to be better suited for quantification purposes due to its simplicity of use and to the wide range of applications that are made possible by technical improvements.

## Conclusion

Our data show that the taxane-induced cell cycle block begins in the first few hours after DOC treatment, whereas at that time only a minority of cells display a decrease in Δψ_m_. These events suggest that the actual impairment in the mitochondrial function, which initiates the apoptotic process, has a later onset and follows the cell cycle block. However, as already highlighted, this impairment is of great importance because, over time, the near totality of DOC-treated cells that presented a loss in Δψ_m_eventually undergoes apoptosis.

By evaluating different cytometric methods concomitantly, we were able to draw up a chronological table of apoptotic events in taxane-induced cell death, where G_2_/M arrest appears to precede mitochondrial involvement, which occurs before DNA fragmentation and cell surface modifications. The present study also provides a sort of time-based guideline for the choice of the most appropriate parameters to detect specific apoptotic modifications, such as cell surface changes or early/initial *vs*. late/massive DNA fragmentation.

In conclusion, we believe that the effects of a particular drug on a cell culture can best be investigated by using multiple parameters to assess apoptosis, by following them up for a relatively long time, and by comparing the dynamics of treated and untreated cell populations. Having said that, some information may still be missing, such as the *absolute *number of disintegrated cells, as there is no way of relating the amount of cell debris to that of the cells which originated them. On the other hand, a fairly simple assay such as cell cycle distribution and its evolution over time may be sufficiently informative to determine whether or not a drug warrants further investigation.

## Methods

### Materials

Unless otherwise specified, all reagents were purchased from Sigma-Aldrich (St. Louis, MO, USA).

### Cell lines

Most studies were performed on a bladder carcinoma cell line, HT1376, obtained from the American Type Culture Collection (Rockville, MD, USA). Cells were maintained as a monolayer at 37°C in a humidified atmosphere of 5% CO^2 ^in air and subcultured weekly. Culture medium was composed of DMEM/HAM F12 (1:1) supplemented with foetal calf serum (10%), glutamine (2 mM), non-essential aminoacids (1%) (Mascia Brunelli s.p.a., Milan, Italy), and insulin (10 mg/mL). Cells were used in the exponential growth phase in all the experiments.

Some experiments were performed on U937 myelomonocytic cell line, obtained from the same source. Cells were grown as liquid cultures at 37°C in a humidified atmosphere of 5% CO_2 _in air and subcultured every three days. Culture medium was RPMI 1640 (Biowhittakers, Verviers, Belgium) supplemented with foetal calf serum (10%), glutamine (2 mM), penicillin (100 IU/L), and streptomycin (0.1 g/L).

### Cell treatments

DOC was obtained from Aventis Pharma S.A. (Paris, France). The drug was stored at a concentration of 10 mg/mL (12.6 mM) in 13% w/w ethanol at 4°C and diluted in medium before use. The final concentration of ethanol in the medium never exceeded 0.01%, and therefore had no effect on cell growth and viability. Control cells received the same amount of solvent.

After performing an initial evaluation of drug cytotoxicity by treating the cell cultures with DOC at concentrations ranging from 0.03 to 3 μg/mL, the highest dose was used in all subsequent experiments.

### Chemosensitivity assay

The Sulforhodamine B (SRB) assay was used according to the method by Skehan et al. [37]. Briefly, cells were collected by trypsinisation, counted and plated at a density of 10,000 cells/well in 96-well flat-bottomed microtitre plates (100 μl of cell suspension/well). In the chemosensitivity assay, DOC was tested at scalar concentrations ranging from 0.03 μg/mL to 3 μg/mL (3.7 μM) for 24 hours or for 1 hour followed by 23-hour culture in drug-free medium. Experiments were run in octuplet, and each experiment was repeated three times. The optical density (OD) of treated cells was determined at a wavelength of 540 nm by means of a fluorescence plate reader. Growth inhibition and cytocidal effect of the drug were calculated according to the formula reported by Monks et al. [38]: [(OD_treated _- OD_zero_)/(OD_control _- OD_zero_)] × 100%, when OD_treated _is > to OD_zero_. If OD_treated _is above OD_zero_, treatment induced a cytostatic effect, whereas if OD_treated _is below OD_zero_, cell killing has occurred. The OD_zero _depicts the cell number at the moment of drug addition, the OD_control _reflects the cell number in untreated wells and the OD_treated _reflects the cell number in treated wells on the day of the assay. In this way, if a drug does not affect cell growth or viability, the test outcome will be 100. Conversely, if it induces a cytostatic effect, the result will be between 0 and 100 (upper part of the diagram), whereas if it induces a cytocidal effect, the result will be between 0 and -100 (lower part of the diagram), indicating that the number of cells at the end of the observation time is lower than the initial one.

### Induction of apoptosis

Induction of apoptosis was tested at a concentration of 3 μg/mL (3.7 μM) DOC for 1 hour followed by a 6-, 24-, 48- 72- and 96-hour culture in drug-free medium. Data are the average of two to three experiments, with errors under 10%.

### Cyclosporin A treatment

Cyclosporin A (CsA) is the most widely used blocker of calcium efflux from the mitochondria. For this preliminary experiment we used a concentration of 1 μM [39]. CsA was added to HT 1376 cells 30 min before DOC treatment. After a 1-hour incubation, both drugs were washed out. Evaluation of JC-1, AnnV and PI staining was carried out immediately after the end of drug exposure and after a 16- and 24-hour culture in drug-free medium. Control (untreated) and DOC-treated cultures were run in parallel and evaluated at the same times as CsA+DOC-treated cultures.

### Flow cytometry

For cytofluorimetric evaluations, the medium was removed at the indicated wash-out times and cells were detached from the flasks by trypsin treatment, washed twice with PBS and stained according to the different methods specified below. A FACS Vantage flow cytometer (Becton Dickinson, San Diego, CA, USA), equipped with an argon laser (488 nm) was used. Data acquisition and analysis were performed using CELLQuest Pro software (Becton Dickinson, San Diego, CA, USA).

### Cell cycle distribution

Cell cycle distribution and percentage of cells within the sub-G1 peak (i.e. with fragmented DNA) were estimated by PI staining. Cells (1 × 10^6^/mL) were fixed in 70% ethanol, washed twice in PBS, then incubated overnight at 4°C in the dark in a PBS solution containing propidium iodide (10 μg/mL), RNAse (10 Kunits/mL) and NP40 (0.01%). Data were elaborated using Modfit software (DNA Modelling System, Verity Software House, Inc., Topsham, ME, USA) and expressed as fractions of cells in the different cycle phases. Samples were run in triplicate, and each experiment was repeated three times. Diagrams of growing cultures display the characteristic x-axis distribution according to the DNA content, the first peak corresponding to the diploid (2c) peak, i.e. to cells in the G_0/1 _phase, and the second peak to cells with 4c DNA content, i.e. to cells in G_2_/M phase. Cells with an intermediate DNA content are in the S phase. When DNA is fragmented, as in apoptotic cells, the affinity with the intercalating PI dye is decreased and a so-called hypodiploid peak (or area) becomes apparent to the left of the G_0/1 _peak.

### JC-1

The cationic fluorochrome JC-1 (BD Biosciences Pharmingen, San Diego, CA, USA) was used to evaluate the decrease in transmembrane mitochondrial potential (Δψm) that accompanies the efflux of pro-apoptotic molecules fromthe mitochondrion. JC-1 forms aggregates that fluoresce red in the presence of high mitochondrial membrane potential, as in normal cells. When mitochondrial membrane potential decreases, as occurs when mitochondrial pores open during the apoptotic process [8,9], JC-1 becomes monomeric and fluoresces green.

After DOC treatments, cells were harvested, washed once in PBS and then immediately incubated in JC-1 working solution containing 1 μM JC-1 monomer at a concentration of 1 × 10^6 ^cells/mL for 15 min in a humidified atmosphere at 37°C in the dark, according to the manufacturers' instructions. Cells were then washed twice, resuspended in Assay Buffer and analysed by flow cytometry. For each sample, 15,000 events were recorded.

### Propidium iodide staining

The DNA-intercalating dye, propidium iodide, does not permeate viable cells. Therefore, when added to unpermeabilised cells, it is possible to assess the amount of necrosis in a cell population. When used in combination with a marker of apoptosis, it permits detection of so-called "secondary necrosis" or "late apoptosis", i.e. the phenomenon whereby apoptotic cells, over time, lose their membrane integrity. Thus, cells that are stained by PI alone have undergone *bona fide *necrotic cell death, whereas PI+ cells that are also stained with a marker of apoptosis can be classified as apoptotic. PI was added to a final concentration of 5 μg/mL immediately before cytometric evaluation (longer incubation times may cause aspecific staining).

### Lectin staining

MAA lectin binds to 2,3-linked syalil residues on cell surface. Cells (5 × 10^5^) were stained for 15 min in the dark at room temperature with 1.5 μg FITC-conjugated MAA lectin [40] dissolved in 100 μL PBS. Cells were thenwashed twice in PBS and fixed in a 0.74% solution of formaldehyde in PBS. Immediately before cytometric analysis, propidium iodide was added to a final concentration of 5 μg/mL to distinguish between early (PI-) and necrotic or late (PI+) apoptotic cells. For each sample, 15,000 events were recorded.

### Annexin V assay

Annexin V binds to phosphatidylserine, a lipid component of the plasma membrane that is flopped from the inner to the outer cell surface during apoptosis and is believed to play a role as an "eat-me" signal [41].

After DOC treatments, cells were harvested, washed once in PBS and incubated with 5 μl/mL Annexin V-FITC in binding buffer (Bender MedSystems, Vienna, Austria) for 10 min at room temperature in the dark. Cells were then washed in PBS and suspended in binding buffer. Immediately before flow cytometry analysis, propidium iodide was added to a final concentration of 5 μg/mL to distinguish between early (PI-) and necrotic or late (PI+) apoptotic cells. For each sample, 15,000 events were recorded.

### TUNEL assay

In the TUNEL assay, 3' OH termini, resulting from DNA cleavage, are labelled with FITC-conjugated nucleotides in a reaction utilsing exogenous terminal deoxynucleotide transferase (TdT).

After DOC treatments, cells were harvested, fixed in 1% paraformaldehyde in PBS on ice for 15 min, suspended in ice cold ethanol (70%) and stored overnight at -20°C. Cells were then washed twice in PBS and incubated with 50 μl of solution containing TdT and FITC-conjugated dUTP (Roche Diagnostic GmbH, Mannheim, Germany) in a humidified atmosphere for 60 min at 37°C in the dark. Samples were then washed in PBS containing Triton X-100 (0.1%), counterstained with 3 μg/mL propidium iodide and RNase (10 Kunits/mL) for 30 min at 4°C in the dark, and finally analysed by flow cytometry. For each sample, 10,000 events were recorded.

### Cell count and viability assay

HT1376 cells were seeded in duplicate at a concentration of 2 × 10^5 ^cells/ml in 35-mm well dishes. At the indicated times, cells were detached by trypsin treatment, washed and resuspended in PBS. An aliquot of the cell suspension was combined with an equal volume of 0.4% Trypan Blue and incubated for 8–10 min. at 37°C. Cells were counted in a counting chamber (Kova Glasstic Slide Hemocytometer, Hycor Biomedical Inc., Garden Groove, USA). Both total cell count and the percentage of viable and non viable cells were recorded. Cells are considered to be viable when they exclude the dye.

### DNA gel electrophoresis

In the course of the apoptotic process, DNA is cleaved in a distinctive way at internucleosomal sites by a specific caspase-activated endonuclease [42], thus yielding fragments in multiples of 200 bp, which appear as a characteristic "ladder" when DNA is separated by gel electrophoresis.

Fragmented DNA was isolated from samples of 2 × 10^6 ^cells as described by Zhivotovsky [42]. Electrophoresis was carried out in 1.5% agarose gel.

### Morphological investigation

After DOC exposure, samples were immediately stained with the cationic fluorochrome JC-1 as described above and with the cell-permeable dye 4',6-DAPI 10 μL/mL (Molecular Probes, Leiden, The Netherlands). Cells were fixed in ethanol (70%) and then examined under a fluorescence photomicroscope (Zeiss, Axioscope 40) to visualise the mitochondrial membrane depolarisation and concurrently the chromatin condensation and/or fragmentation typical of apoptotic cells.

## List of abbreviations used

DOC, docetaxel; SRB, Sulforhodamine B; MAA, *Maakia Amurensis *lectin; AnnV, Annexin V; PI, propidium iodide; PS, phosphatidylserine; mPTP, mitochondrial permeability transition pore; PBS, phosphate-buffered saline.

## Authors' contributions

All authors were involved in study design and planning of the experiments, under the supervision of WZ and MM, who acted as scientific advisors. FF was responsible for coordinating the laboratory work and for data analysis; he also performed most of the cytometry analysis. SC, GB and RL performed the *in vitro *experiments. MM was responsible for drafting the paper. All authors read and approved the final manuscript.
